# Immune Signaling and Antimicrobial Peptide Expression in Lepidoptera

**DOI:** 10.3390/insects4030320

**Published:** 2013-07-02

**Authors:** Ángel M. Casanova-Torres, Heidi Goodrich-Blair

**Affiliations:** Department of Bacteriology, University of Wisconsin-Madison, WI 53706, USA; E-Mail: amcasanova@wisc.edu

**Keywords:** Lepidoptera, recognition, signaling, antimicrobial peptide, pest control

## Abstract

Many lepidopteran insects are agricultural pests that affect stored grains, food and fiber crops. These insects have negative ecological and economic impacts since they lower crop yield, and pesticides are expensive and can have off-target effects on beneficial arthropods. A better understanding of lepidopteran immunity will aid in identifying new targets for the development of specific insect pest management compounds. A fundamental aspect of immunity, and therefore a logical target for control, is the induction of antimicrobial peptide (AMP) expression. These peptides insert into and disrupt microbial membranes, thereby promoting pathogen clearance and insect survival. Pathways leading to AMP expression have been extensively studied in the dipteran *Drosophila melanogaster*. However, Diptera are an important group of pollinators and pest management strategies that target their immune systems is not recommended. Recent advances have facilitated investigation of lepidopteran immunity, revealing both conserved and derived characteristics. Although the general pathways leading to AMP expression are conserved, specific components of these pathways, such as recognition proteins have diverged. In this review we highlight how such comparative immunology could aid in developing pest management strategies that are specific to agricultural insect pests.

## 1. Introduction

Lepidoptera include agricultural pests that, through feeding and other activities, negatively affect stored grains [1], food and fiber crops [2,3]. Since a single Lepidoptera adult can produce hundreds of eggs, and their primary food source is typically plant material, they can cause significant damage to agricultural crops. Although biological agents can help manage these insect pests, insecticides currently are essential for large-scale effective and economical pest control [4]. These insecticides can also affect non-target organisms, including pollinators, and their application not only disrupts natural ecosystems but also reduces yields of crops that rely on pollination [5,6]. The non-target effect of some pesticides is in part due to their effects on insect immunity, which is necessary for insect survival in natural environments. For example, currently used pesticides have been shown to affect cellular [7,8,9,10] and humoral [11,12] immune responses and interfere with grooming behavior [13,14]. These effects on immunity are likely non-specific and negatively impact the health of both the target pest and beneficial arthropods. Therefore, there is a need for novel target-specific approaches to control insect pests without affecting beneficial arthropods.

Although immune pathways can be generally and non-specifically inhibited by pesticides, they also are a likely source of candidate molecules that could be inhibited for target-specific insect control since multiple classes of insect immunity genes, including signaling pathways, can be under strong selection for diversification [15]. Fundamental mechanisms of innate immunity comprising cellular and humoral pathways are conserved throughout the animal kingdom [16] and are controlled by signaling pathways activated by various stimuli [17,18], including pathogen recognition by immune surveillance systems. Despite this overall conservation, aspects of immune systems are subject to strong selection to evolve in response to varying pathogen exposure and to pathogen evolution of virulence determinants that modulate immunity [15,19,20,21]. Such co-evolutionary dynamics can promote diversification of conserved elements of immunity as well as the recruitment of novel effectors [22]. As such, the investigation of insect immune pathways and mechanisms of pathogen modulation can yield insights into components that may be susceptible to inhibition. For example, the insect pathogen *Xenorhabdus nematophila* suppresses cellular and humoral immunity in the lepidopteran moths *Manduca sexta* and *Spodoptera exigua* [23,24] but not in the dipteran fly *Drosophila melanogaster* [25], suggesting the stage of immunity suppressed by *X. nematophila* may be absent from *D. melanogaster*, but present in Lepidoptera. Since dipteran flies serve as pollinators [26,27], decomposers, food sources for other animals, and pest control agents, capitalizing on the possible differences between dipteran and lepidopteran immune signaling cascades will help in the identification of targets for pest-specific inhibition. With this knowledge in hand, pest management can be achieved by developing small molecule inhibitors of these targets that will suppress pest insect immunity and lead to increased susceptibility to environmental pathogens. Indeed, many insecticides may contribute to insect (target and non-target) death by modulating aspects of immunity [5]. The feasibility of targeted pest control via insect immune inhibition has been established for termites; a small molecule inhibitor of an immune surveillance protein led to faster termite death upon exposure to various pathogens [28].

Much of our current knowledge of insect immune signaling pathways and receptor and effector function is based on the premiere model organism *D. melanogaster*, for which there are extensive genetic tools and several fully sequenced genomes [29]. Well-established lepidopteran insect models such as the silkworm *Bombyx mori* and the tobacco hornworm *M. sexta* also have been widely used to study insect immunity. These organisms have been particularly useful for investigating hemolymph proteins and hemocyte function because of their relatively large larval size and hemolymph volume [16]. Many insects in the order Lepidoptera are easy to rear in laboratory conditions, and new tools such as RNA interference have been implemented successfully to study genetics of their immune systems [30,31]. Also, their immune signaling pathways are gradually being revealed by genomic and transcriptomic data [32,33,34,35,36,37,38]. Based on these model insect systems a fairly detailed picture of immunity, from pathogen detection to effector function, is emerging, though many gaps remain, particularly with regard to components that are unique to different insect orders. Here we review aspects of insect immunity with an emphasis on the similarities and distinctions between *D. melanogaster* and representative Lepidoptera.

## 2. Insect Immunity

In insects, the cellular immune response includes phagocytosis, nodulation and encapsulation [39,40,41,42] and the humoral response involves the expression of antimicrobial peptides (AMPs) [43,44] as well as the pro-phenol oxidase (proPO) proteolytic cascade that results in formation of melanized nodules and toxic reactive compounds [45,46]. AMPs are small cationic peptides that insert into and disrupt microbial membranes, thereby killing and clearing pathogens [44]. They are synthesized by hemocytes and to a greater extent in fat body from which they are released into the insect hemolymph rapidly after microbial infection [43,47]. AMPs are also expressed in extra-embryonic tissues of eggs [48], which may help protect the developing embryo from infection.

AMPs are a conserved component of immunity in plants [49] and animals [50] and while they have diverse structures most can be assigned to larger families such as cecropins, attacins, defensins and diptericins [51]. Their diversity and immune effector function as well as their variant representation among insects (Table 1) have made them a central focus in the study of invertebrate pathology [30,52]. In *D. melanogaster* AMP synthesis is transcriptionally regulated through the Toll and immune deficiency (IMD) pathways. Each of these pathways is activated by detection of microbial components via different pattern recognition receptors (PRRs) that trigger, through complex regulatory cascades, nuclear factor kappa B (NF-κB) dependent transcription of the genes encoding AMPs. After AMPs are translated in the cytoplasm they are released into the hemolymph where their high concentrations and broad activity are thought to enhance clearance of invading microorganisms from the insect [53]. Bioinformatic and experimental data support the existence of the AMP-inducing Toll and Imd pathways in lepidopterans, though not all components have been identified in model organisms such as *M. sexta* [32,35]. The conserved presence of AMPs in immunity coupled with the possibility that certain elements of their induction pathways may vary among insects enhances the probability that microbially-induced AMP expression could be inhibited in a pest-specific manner. As such, for the remainder of this review we focus on pathways leading to AMP gene expression. 

**Table 1:** Lepidoptera-specific immune effectors.

Receptor/Effector	Class	Activity	Reference
**BmPGRP-L1-L5**	PRR	Peptidoglycan recognition	[54,55]
**βGRP1-3**	PRR	β–1,3 glucan recognition	[54,56,57]
**Hemolin**	PRP	Binds LPS and LTA; triggers cellular response	[58,59]
**HP14**	PRR	Binds Lys-PGN; triggers proPO activation	[60]
**Moricin**	AMP	Antibacterial activity against Gram-positive and Gram-negative bacteria; targets cytoplasmic membrane; increases membrane permeability	[61]
**Gloverin**	AMP	Antimicrobial activity against fungi, and Gram-negative and Gram-positive bacteria; targets outer membrane; inhibition of outer membrane proteins	[62]
**Lebocin**	AMP	Antimicrobial activity against fungi, and Gram-negative and Gram-positive bacteria	[63,64]

## 3. Signaling Pathways Involved in Antimicrobial Peptide Gene Expression

### 3.1. Induction of AMP Genes by the NF-kB Family of Transcription Factors

In *D. melanogaster,* transcription of AMP-encoding genes is activated by the NF-κB family transcription factors in response to infection [65,66,67,68,69] with distinct NF-κB family transcription factors responding to the Toll and immune deficiency (IMD) signal transduction pathways [70]. In response to Toll pathway activation, the NF-κB inhibitor Cactus is phosphorylated and degraded allowing its targets, the NF-κB factors Dif and Dorsal, to be translocated to the nucleus. IMD pathway activity culminates in the NF-κB factor Relish being activated by a stimulus-induced proteolytic cleavage [71]. In the case of Dif and Dorsal, gram-positive bacterial and fungal infections primarily serve as the stimuli that induce degradation of Cactus through the Toll signaling pathway. In general, gram‑negative bacterial infections of *D. melanogaster* stimulate the proteolytic cleavage of Relish through the IMD pathway. Once in the nucleus, these transcription factors drive the transcription of immune effectors, including AMP genes whose promoters contain NF-κB binding sites [70,72]. Overall, NF-κB proteins and their DNA-binding specificities are conserved among organisms, including those Lepidoptera studied to date [73,74]. However, the NF-κB-binding regions for Inhibitor of κB (IκB) proteins (e.g., Cactus) are not conserved, suggesting diversification and co-adaptation between IκB and NF-κB pairs [74]. Also, recent work indicates that NF-κB nuclear co-regulators may contribute to species-specific regulation of AMP gene expression [75]. Therefore, modulation of inhibitors and nuclear-co-regulators of NF-κB-dependent transcription may be one avenue by which target-specific immune suppression could be achieved. 

### 3.2. Recognition and Proteolytic Cascades

In *D. melanogaster*, NF-κB-dependent AMP induction through the Toll and Imd pathways is activated by detection of microbial components via different pattern recognition receptors (PRRs). PRRs are soluble or membrane-bound proteins that bind to specific microbe associated molecular patterns (MAMPs) such as lipopolysaccharide (LPS), lipoteichoic acid (LTA), peptidoglycan (PGN) or β-1,3-glucan that are released from or are found on the cell surfaces of bacteria or fungi [55]. Upon interaction with MAMPs, PRRs can directly agglutinate pathogens or trigger proteolytic signaling cascades and cytokine release, which in turn lead to the activation of downstream cellular and humoral pathways, including pro-PO activation and AMP gene expression [16,65,76].

PGN recognition proteins (PGRPs) and β-1,3-glucanase-related proteins (βGRPs) were discovered in the lepidopteran silkworm (*B. mori*) by assaying for plasma components that activate the proPO cascade [77]. PGRPs were subsequently shown to be conserved across mammals and insects [78], and in *D. melanogaster* their role in the induction of AMP gene expression through Toll and IMD pathways in response to PGN has been well documented [79,80,81,82,83]. Similarly, βGRPs have been shown to induce AMP expression through Toll pathway in response to fungal infections [79,84]. In contrast, there is a dearth of literature linking specific PGRPs or βGRPs to AMP induction in Lepidoptera [85]. Such a link is possible, since PGN and β-1,3-glucan can activate AMP gene expression in *M. sexta* and *B. mori* [85,86,87,88,89,90] and multiple infection-induced PGRP- and βGRP-encoding genes have been identified in Lepidoptera [32,38,54,55,91,92,93,94]. However, there are numerous hints that Lepidoptera and Diptera may have evolved divergent mechanisms of linking pathogen detection to conserved Toll and IMD signal transduction cascades. First, a genome comparison between *B. mori* and *D. melanogaster* failed to identify 1:1 PGRP orthologs [54]. Similarly, *B. mori* gram-negative binding protein (GNBP) and *M. sexta* microbe binding protein (MBP), members of the β-1,3-glucanase-related protein superfamily [76,95], appear to be distantly related to *D. melanogaster* GNBPs [76], suggesting divergence of this group of proteins. *M. sexta* MBP expression is strongly up-regulated in fat body after immune challenge and shows specific binding to LTA, LPS, DAP-PGN [76]. Also, in contrast to the situation in *D. melanogaster*, highly purified LPS and LTA are inducers of AMP gene expression in Lepidoptera, though not as potently as crude LPS (with contaminating PGN) or purified PGN [85,90,96,97]. This raises the possibility that different MAMPs or combinations of MAMPs are most efficacious in eliciting AMP gene expression in Lepidoptera relative to Diptera. Also, since purified LPS can elicit AMP expression in Lepidoptera but not *D. melanogaster*, Lepidoptera have either a distinct repertoire of PRRs responsible for LPS-dependent triggering of Imd or Toll pathways, or an as-yet undiscovered pathway that links LPS to AMP induction. Testing these ideas awaits the identification of the suite of PRRs and signal transduction pathways responsible for transducing LPS, LTA, PGN, or combinatorial microbial signals to AMP gene expression. 

One class of lepidopteran PRR that may mediate infection-dependent induction of AMPs is the C‑type lectins (CTLs), Ca^2+^-dependent, secreted proteins that have carbohydrate-binding capabilities. Lepidopteran CTLs are involved in immunity. Similar to some CTLs of *D. melanogaster* [98], several CTLs of *M. sexta* [55] and *B. mori* [54,99] are reported to mediate induction of cellular responses and the proPO cascade. Although the nomenclature quickly becomes confusing, CTLs include lipopolysaccharide-binding protein (LBP)*. B. mori* LBP binds LPS and triggers cellular responses (nodulation) [100]. Finally, immulectins (IML) are also CTLs. *M. sexta* IML-1 binds to Gram-positive and Gram-negative bacteria as well as yeast [101], IML-2 shows specific binding to LPS [102], IML-3 and IML-4 show specific binding to LPS and LTA, and IML-3 can also bind laminarin, a ß-1,3-glucan [103,104]. Diversity in CTL carbohydrate-binding specificities may result in lineage-specific pathogen recognition-signal transduction connections.

Of particular relevance to the theme of this review are PRRs present in Lepidoptera but not Diptera (Table 1). In general, both orders of insects encode βGRPs and PGRPs. However, specific representatives of each class are restricted to Lepidoptera (Table 1). For example, the Lepidopteran βGRP-2, which binds fungal cell wall β-1,3 glucans [55] and LTA [105], is absent from Diptera [54]. Such derived βGRP and PGRPs may contribute to lepidopteran-specific transduction of signals to downstream pathways. Other Lepidoptera-specific PRRs are hemolin and hemolymph proteinase-14 precursor (proHP14) (Table 1). Like IML C-type lectins, hemolin is an LPS- and LTA-binding PRR [58] with roles in mediating cellular responses and as an opsonin to enhance phagocytosis [59]. HP14 has been shown to detect and bind a broad range of MAMPs, and may coordinate with βGRP1 or βGRP2 to activate proPO [60,106]. The potential role of the PRRs discussed above in mediating the expression of AMP genes remains to be determined, and further study of the Lepidoptera-specific immune surveillance proteins and divergent activities of conserved PRRs likely will yield novel avenues for pest-control. 

### 3.3. Toll Pathway

**Figure 1:**
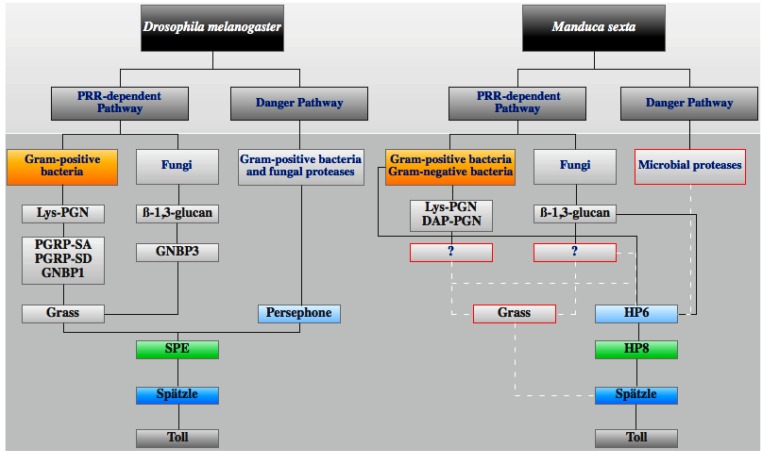
Toll-activating signal transduction pathways in *D. melanogaster* and *Manduca sexta*. (**A**) The *D. melanogaster* Toll pathway based on the revised model presented in Ashok 2009 [107]. MAMP/PRR-dependent or a MAMP-independent danger signal cascades can both activate Toll. (**B**) Current knowledge of the *M. sexta* Toll pathway. The *M. sexta* Toll pathway can be activated by MAMPs, but the specific PRRs and proteolytic cascade(s) responsible for this activation have not been reported. Known components of Spätzle-activation include the proteases HP6 and HP8, which are most closely related to the danger-pathway proteases of *D. melanogaster*. However, the induction of the Toll pathway by MAMP-independent signals has not been documented yet in *M. sexta*. Dashed lines indicate potential pathways that have not been experimentally proven in *M. sexta*. Orange boxes represent key differences between *D. melanogaster* and *M. sexta* Toll pathway; a red border highlights proteins or pathways not yet identified in *M. sexta*. Boxes with same color are for putative orthologous steps. PGRP, peptidoglycan recognition protein; GNBP, gram negative binding protein; SPE, Spätzle processing enzyme; HP, hemolymph proteinase.

*D. melanogaster* has both MAMP-dependent and MAMP-independent routes to activate the Toll pathway [107]. In MAMP-dependent Toll induction, bacterial Lys-PGN (typical of gram-positive bacteria) is detected by PGRP-SA or PGRP-SD (in the presence of GNBP-1), while yeast or fungal β‑1,3-glucan is detected by GNBP-3 [85,108,109] (Figure 1). MAMP-independent stimuli are virulence determinants, such as proteases and chitinases, secreted by microbes and dubbed “danger signals” [110]. MAMPs and MAMP-independent stimuli each trigger a distinct proteolytic cascade [111] that both culminate in cleavage of the cytokine Spätzle by the serine protease Spätzle processing enzyme (SPE) [112]. Interaction of active Spätzle C-terminal domain (C-106) with the surface‑localized Toll receptor triggers an intracellular signal transduction terminating in induced expression of AMPs and cellular responses [113]. 

**Figure 2:**
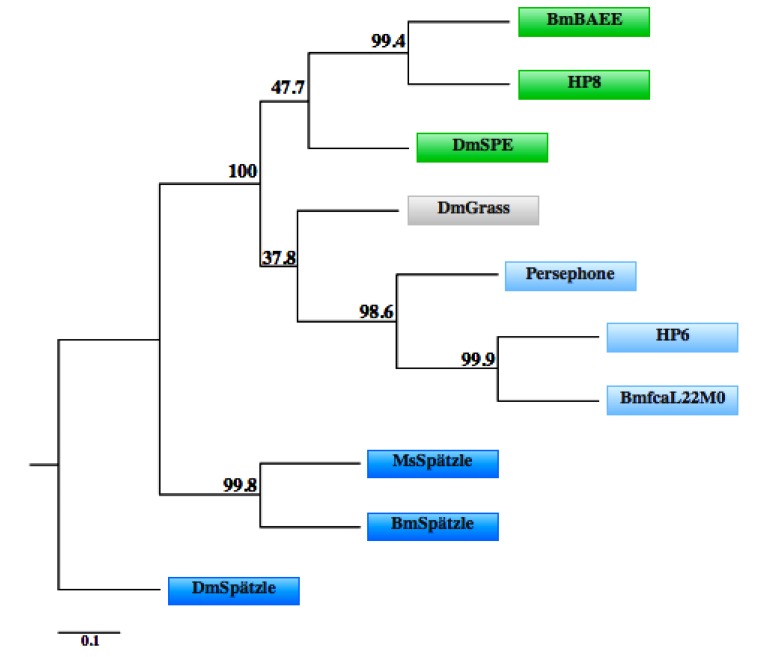
Phylogenetic relationships among serine proteases involved in Toll pathway activation. Sequence alignment and tree construction were performed using the amino acid sequence of *D. melanogaster* (Dm) Grass (Q86PB3), SPE (NP_651168.1), Persephone (Q9VWU1) and Spätzle (NP_524526.1); *M. sexta* (Ms) HP6 (AAV91004.1), HP8 (AAV91006.1) and Spätzle (ACU68553.1); and *B. mori* (Bm) fcaL22M01 (AK384444), BAEE (H9J6N1), and Spätzle (NM_001114594). Bm serine proteases were identified by TBLASTN [120] analysis the Bm genome for homologs of Ms serine proteases. The roles of these Bm proteins in Toll pathway activation have not been shown experimentally. One thousand bootstrap repetitions were performed to estimate the reliability of the tree; the percent values obtained are indicated on the nodes. Sequence alignment was performed using Clustal Omega [121,122] bootstrapping analysis, matrix calculation, matrix transformation were conducted by the Fitch-Margoliash method and the combination of the 1,000 resulting trees was identified using the Seqboot, Protdist, Fitch and Consense programs within the Phylip phylogenetic analysis package [123]. The phylogenetic tree was constructed using Phylodendron software version 0.8d, by D.G. Gilbert [124]. The phylogenetic relationships observed are consistent with those previously published [116,119]. Colored boxes for each protein match those presented in Figure 1.

Some of the basic components of the Toll pathway are present in Lepidoptera (Figure 2). *M. sexta* hemocytes express an infection-induced Toll-like receptor and the genome of *B. mori* encodes 14 genes predicted to encode Toll-like receptors as well as homologs of each of the intracellular components of Toll-dependent signaling [85,114,115]. Both *M. sexta* and *B. mori* encode homologs of the *D. melanogaster* Toll-activating cytokine Spätzle [116] (Figure 2). Also, for both *M. sexta* and *B. mori* there is experimental evidence linking the Toll pathway with AMP induction [116,117,118]. In *M. sexta*, Toll pathway results in expression of several antimicrobial peptides, including attacin-1, cecropin-6, moricin and lysozyme. In addition, the transcript level of hemolin, a pattern recognition protein exclusive to lepidopterans (Table 1), is induced by injection of activated Spätzle-C108 into larvae [116].

Despite the conservation of certain aspects of the Toll pathway, the extracellular cascades that lead to Spätzle activation may have diverged between *D. melanogaster* and the two Lepidoptera (Figure 1). For example, in contrast to what is known in *D. melanogaster*, the *M. sexta* Toll pathway is activated by gram-negative-associated MAMPs [115,118]. Also, the genome of *B. mori* lacks 1:1 orthologs of Grass, Spirit and Persephone [54], the *D. melanogaster* serine proteases responsible for MAMP/PRR-dependent and MAMP-independent cleavage of SPE (Figure 1). Progress has been made in identifying a *M. sexta* proteolytic cascade that results in processing pro-Spätzle into its active C-terminal domain (C-108). The direct cleavage is mediated by hemolymph proteinase (HP) 8 [11,13], a homolog of *D. melanogaster* SPE [119], In turn, HP8 is processed into its active form by HP6. HP6 is most similar to *D. melanogaster* Persephone protease, which activates SPE in response to MAMP-independent stimuli [110,113]. This hemolymph proteinase is activated in response to Gram-positive or Gram‑negative bacteria and in response to ß-1,3-glucan [119]. However, the PRRs and proteolytic cascade that transduce MAMP signals to AMP induction are unknown (Figure 1). 

The findings reviewed above demonstrate that while the overall architecture of the Toll pathway is conserved among insects, the specific identities of proteolytic cascade members are distinct and many gaps remain in our understanding of Toll activation in Lepidoptera. Filling these gaps should reveal potential lineage-specific molecules that can serve as targets to hinder the activation of the Toll pathway in agricultural pests.

### 3.4. IMD Pathway

In *D. melanogaster* the IMD pathway also contributes to AMP gene induction and is triggered by direct interaction of DAP-PGN, a MAMP typical of gram-negative bacteria, with the transmembrane receptor PGRP-LC [82,83,125,126]. Other members of the PGRP family also play a role upstream of IMD. For example, PGRP-LE can act as an intracellular receptor for monomeric PGN [127] and its truncated form can enhance PGRP-LC-mediated recognition [128]. DAP-PGN/PGRP-LC interaction activates intracellular IMD, which then recruits FAS-associated death domain (FADD) and death‑related ced-3/Nedd2-like protein (Dredd) to form a complex [129,130]. Current evidence supports the idea that Dredd, a caspase-like molecule, cleaves the NF-κB transcription factor Relish [131]. Imd also appears to activate a phosphorelay: the transforming growth factor-ß (TGFß)-activated kinase 1 (TAK1) phosphorylates the IkB kinase (IKK) “signalosome”, which in turn phosphorylates Relish and contributes to its cleavage [131]. Relish cleavage into its activated amino-terminal transcriptional regulator domain allows its translocation into the nucleus, where it activates AMP gene expression [71]. The translocation of Relish into the nucleus is regulated by two recently discovered components of this pathway: inhibitor of apoptosis 2 (Iap2) and transforming growth factor-activated kinase 1 (TAK1)‑binding protein 2 (Tab2) [132,133]. Both, Iap2 and Tab2 act upstream of Relish and downstream of IMD, while Iap2 functions downstream of TAK1 [132,134]. Of particular importance to AMP gene expression is Iap2, the knockdown of which hampers the sustained expression of AMP genes [135]. While Iap2 and Tab2 are necessary for Imd signal transduction, the gene product of *pirk*, a recently characterized gene, interacts directly with IMD and PGRP-LC. Pirk overexpression analyses revealed that it acts as a negative regulator by reducing the expression of the AMP genes attacin B, cecropin B, and diptericin B, which are all under the control of the Imd pathway [136].

Most of the information available about the Imd pathway in Lepidoptera comes from bioinformatics; orthologs of all intracellular components of the Imd pathway have been found in *B. mori* [54,137] and *M. sexta* [35]. However, few experiments have been done to characterize the molecular mechanisms leading to activation of the Imd pathway in these insects. In *M. sexta* several genes of the Imd pathway, including those encoding IMD, FADD, TAK1, Dredd and Relish are up regulated in fat body of immune challenged 5th instar larvae [35] and in the midgut of *B. mori* during the wandering stage [36]. Genes encoding lysozyme, moricin and defensin AMPs also were up regulated in the midgut of *B. mori* in the wandering stage [36], consistent with the possibility that AMP induction is IMD-mediated. Finally, in the lepidopteran beet armyworm *Spodoptera exigua*, RNAi-mediated knockdown of Relish expression resulted in loss of cecropin induction upon fungal infection [138], further strengthening the idea that the IMD pathway may contribute to AMP gene expression in Lepidoptera, though perhaps it is triggered by distinct signals. Further study is needed to elucidate IMD-mediated AMP induction in Lepidoptera and to reveal any differences there are in this pathway between Diptera and Lepidoptera.

## 4. Conclusions

Insecticides are necessary to guarantee effective insect pest management in agricultural settings. However, the cost and off-target effects of these insecticides directly and indirectly increase economic burden; the latter by affecting beneficial arthropods such as pollinators. The study of insect immunity can provide tools for the development of target-specific cost-effective approaches to control agricultural pests. Directed suppression of pest immune defenses is predicted to render them susceptible to environmental and applied biocontrol pathogens, as recently demonstrated in termites by Bulmer and colleagues [28]. The studies summarized above suggest that many aspects of insect immunity, including recognition factors and serine proteases, have diverged between *D. melanogaster* and Lepidoptera. Continued comparative immunity of a broad array of species from Diptera, Lepidoptera, and other insect orders will reveal possible candidate immunity factors for target-specific approaches that will enable the effective control of insect pests. However, before such approaches can be realized, the details of lepidopteran immune signaling pathways must be elucidated. The relatively large sizes of last instar larvae of many lepidopteran species will facilitate biochemical approaches to such studies, while the establishment of immune-inducible lepidopteran cell lines such as the UGA-CiE1 cell line [139] can enable the characterization of molecular mechanisms leading to Imd pathway activation and its contribution to AMP gene expression. Finally, ongoing investigations into the immune‑modulatory mechanisms of entomopathogens will help identify key steps in immunity that are susceptible to manipulation, contributing to the development of natural, cost-effective, non-toxic alternatives to chemical insecticides currently used for pest management. 
